# Mindfulness and learning engagement among undergraduate dance majors: the serial mediating roles of self-efficacy and academic burnout

**DOI:** 10.3389/fpsyg.2026.1852116

**Published:** 2026-09-01

**Authors:** Zhihao Gong, Kai Qiu, Feng Xue

**Affiliations:** 1 School of Film and Media, Wuchang College of Technology, Wuhan, China; 2 Department of Dance, Sangmyung University, Seoul, Republic of Korea; 3 Music College, Jimei University, Xiamen, China

**Keywords:** academic burnout, dance majors, learning engagement, mindfulness, self-efficacy

## Abstract

**Background:**

In recent years, China has continued to strengthen arts education reform and the development of aesthetic education. The training regimen for dance majors is characterized by high intensity, high repetition, precise error correction, and continuous assessment; the stress resulting from these factors can affect their physical and mental wellbeing. Understanding the internal mechanism that promotes learning engagement can help clarify the transmission paths among related variables and provide a theoretical basis for targeted intervention strategies.

**Objective:**

This study examines the relationship between mindfulness and learning engagement among undergraduate dance majors, and the mediating effects of self-efficacy and academic burnout.

**Methods:**

The sample comprised 711 undergraduate dance majors from five universities across five provinces in China. Data were collected using the Mindful Attention Awareness Scale, the General Self-Efficacy Scale, the Adolescent Student Burnout Inventory, and the Utrecht Work Engagement Scale-Student. Descriptive statistics and correlation analyses were conducted, and the hypothesized serial mediation model was tested using the PROCESS macro (Model 6) with 5,000 bootstrap resamples.

**Results:**

The findings indicated that (1) mindfulness was significantly and positively associated with learning engagement (r = 0.330, *p* < 0.01); (2) the association between mindfulness and learning engagement was partially mediated by self-efficacy [95% CI = (0.07, 0.17), Boot SE = 0.02]; (3) the association between mindfulness and learning engagement was partially mediated by academic burnout [95% CI = (0.12, 0.22), Boot SE = 0.03]; and (4) the serial indirect effect through self-efficacy and academic burnout was significant [95% CI = (0.04, 0.09), Boot SE = 0.01].

**Conclusion:**

The results validate the sequential mediation roles of self-efficacy and academic burnout in the association between mindfulness and learning engagement. By improving students' mindfulness, strengthening their self-efficacy, and reducing academic burnout, learning engagement in high-standard dance training contexts may be further enhanced.

## 1 Introduction

Learning engagement refers to a relatively enduring and stable state of positive involvement demonstrated by learners in educational activities. It manifests as their dedicated effort and energy invested in learning, their recognition of learning's significance and positive emotional experiences, as well as their high level of focus and immersion in learning tasks (Fredricks et al., 2004; Reschly and Christenson, 2022). Three dimensions are frequently used to conceive it: vigor, dedication, and absorption (Schaufeli et al., 2002). In the world of education, learning engagement has attracted a lot of interest. Researchers frequently regard it as an essential metric for evaluating the quality of education, student growth, and forecasting academic success. It can reflect the true learning state in the classroom. Insufficient learning engagement often manifests as issues such as lack of concentration, academic burnout, decreased enthusiasm for learning, and low classroom participation (Zhang, 2025; Papageorgiou et al., 2025). This is particularly crucial in the context of training under high standards, and in dance education research, it is often regarded as a key indicator of training quality and learning outcomes (Yang and Phee, 2026). Compared with the learning experiences of general university students, dance learning places a stronger emphasis on physicality and performance orientation, and learning outcomes depend heavily on students' sustained physical effort, cognitive processing, and emotional engagement throughout the training process (Schaufeli, 2017; Yang and Phee, 2026). In the context of dance training, vigor refers not only to mental energy but also to physical recovery and training endurance. Dedication reflects not only a recognition of the value of learning but also a commitment to sustained, repetitive practice. Absorption involves not merely maintaining attention but also continuously monitoring bodily sensations, movement details, rhythm, and spatial awareness. Dance training is characterized by “high intensity, high repetition, meticulous correction, and frequent public evaluation,” requiring sustained physical control, sustained concentration, and emotional stability (Dwarika and Haraldsen, 2023). If these pressures continue to build up and result in an undue loss of mental and physical resources, training volume and classroom participation may rapidly decline, thereby diminishing learning engagement. Some studies have also shown a significant correlation between learning engagement and dance performance; increasing learning engagement can help improve various aspects of dance performance (Adem, 2025). Therefore, dance majors form a typical learning context characterized by high academic demands, high exposure to evaluation, and high resource consumption. In this context, examining how psychological resources sustain learning engagement has greater relevance and explanatory value than studies based on general student samples.

### 1.1 Mindfulness and learning engagement

Mindfulness refers to a person's intentional and non-judgmental awareness of present-moment experience (Kabat-Zinn, 2001). From the perspective of its psychological mechanisms, mindfulness helps individuals develop a decentring perspective on the self by enhancing their attention regulation, body awareness, and emotional regulation, thereby promoting higher levels of self-regulation and psychological adaptation (Maloney et al., 2024; Sumińska and Rynkiewicz, 2025). Dance training places high demands on attention regulation, body awareness, and emotional regulation, and these skills are closely linked to the processes involved in mindfulness. It can be inferred that the psychological processes activated by mindfulness are highly consistent with the core abilities needed to maintain technical control and stable performance in dance learning. Related studies have shown that dance learners often display relatively high body awareness and mindfulness tendencies (Fitch and Barnstaple, 2024; Daraki et al., 2024), and mindfulness training has been found to strengthen dancers' psychological resources and improve dance performance (Pandya, 2025).

Academic stress is a persistent and widespread source of significant stress among university students, potentially weakening learning ability and academic performance, reducing motivation, and increasing the risk of dropping out (Pascoe et al., 2020). Previous research has shown a significant positive correlation between mindfulness and learning engagement (Egbaria, 2024; Zhao et al., 2024), and mindfulness practice not only helps improve students' self-regulation and classroom participation (Hammill et al., 2023), but also helps buffer academic stress and burnout to some extent (Chen and Qi, 2025). This suggests that mindfulness may further promote learning engagement by reducing stress burden, improving self-regulation, and maintaining a positive learning state.

For dance learners, high-standard training necessitates a sustained investment of significant physiological and psychological resources (Dwarika and Haraldsen, 2023). Mindfulness may assist learners in maintaining attentional stability and mitigating the interference of negative emotions in high-pressure environments, thereby sustaining a positive learning state (Puhlmann and Engert, 2025; Maloney et al., 2024; Shi et al., 2025). Based on this, this study proposes hypothesis H1: Mindfulness can positively predict learning engagement.

### 1.2 The mediating role of self-efficacy

Bandura's Social Cognitive Theory (SCT) is the source of self-efficacy. It refers to an individual's competence beliefs regarding their ability to organize and execute the behavioral processes necessary to complete specific learning tasks and achieve expected performance (Bandura, 1997). Research has shown that self-efficacy not only stimulates learning motivation but also affects persistence in the face of difficulty, self-regulation, and sustained involvement, thus playing an important role in learning engagement and academic achievement (Zimmerman, 2000; Schunk and DiBenedetto, 2021). Within the Study Demands-Resources (SD-R) framework, self-efficacy can also be viewed as a crucial personal resource that helps individuals maintain a positive assessment of their abilities and a sustained willingness to engage when facing learning tasks, thereby providing a psychological foundation for sustaining motivation.

Existing research provides direct support for the relationship between mindfulness and self-efficacy (Dai, 2024; Abdel Hadi and Al-Quraan, 2025). Mindfulness's emphasis on present awareness and non-judgmental acceptance may help students understand the stress, fatigue, and frustration experienced during training in a more positive and controllable way, thereby increasing their self-efficacy, or their assessment of their own skills and the possibility of finishing the task. At the same time, research has also found a close link between self-efficacy and learning engagement. Self-efficacy can further influence learning engagement through psychological resilience (Wang and Zhang, 2024). When students have strong beliefs about their abilities, they are more likely to set clear goals, exert more effort, and persevere in difficult situations, thus exhibiting a higher level of learning engagement (Zhang, 2025).

For dance students, a stronger sense of self-efficacy may help translate the attentional and emotional benefits of mindfulness into sustained engagement in learning. Existing research in dance education largely remains at the descriptive survey level, with limited modeling of relevant psychological mediating mechanisms, especially a lack of systematic examination of the pathways between mindfulness, self-efficacy, and learning engagement (Dwarika and Haraldsen, 2023). In summary, this study hypothesizes H2: Mindfulness can promote learning engagement by enhancing self-efficacy.

### 1.3 The mediating role of academic burnout

Academic burnout typically refers to a chronic state of physical and mental exhaustion experienced by individuals under prolonged academic pressure, primarily manifested as emotional exhaustion, apathy or detachment from learning, and low self-efficacy (Schaufeli et al., 2002). Similar characteristics have also been observed in high-intensity dance training environments (Longakit et al., 2026; Quested and Duda, 2011).

According to the Study Demands-Resources (SD-R) theory, the accumulation of learning demands can easily trigger negative responses such as fatigue, detachment, and reduced efficacy through an energy-depleting process, while personal resources help buffer the resource consumption caused by high demands, reducing the occurrence and development of burnout. Mindfulness, as an important personal resource, may help buffer the resource consumption caused by high learning demands, thereby reducing academic burnout. A longitudinal study in a school setting showed that high demands are more likely to lead to burnout, while resources are more conducive to maintaining active engagement (Salmela-Aro and Upadyaya, 2014).

Direct evidence already exists that higher levels of mindfulness among university students correlate with lower levels of academic burnout (Gan et al., 2023; Cai et al., 2025a; Liu et al., 2024), and they are less likely to experience fatigue and exhaustion during learning (Cai et al., 2025a). Furthermore, randomized controlled trials have found that mindfulness training can significantly reduce academic stress and burnout among Chinese university students and improve psychological resilience (Chen and Qi, 2025).

In higher education, academic burnout is considered a significant indicator of impaired student wellbeing, closely associated with decreased learning motivation, reduced learning engagement, impaired academic performance, and increased risk of dropping out (Salmela-Aro and Upadyaya, 2014; Madigan and Curran, 2021). According to previous studies, academic burnout and learning engagement are significantly negatively correlated (Mostert et al., 2007). Furthermore, Sun et al. (2025) found that academic burnout is not only a negative outcome variable. However, they may also further erode students' energy investment, concentration, and willingness to continue participating in learning.

In summary, academic burnout may be an important mediating mechanism by which mindfulness further influences learning engagement. We can view academic burnout as a crucial energy hub connecting individual resources and learning engagement. However, existing research has mostly treated academic burnout as an outcome variable or risk indicator, with less emphasis on incorporating it into the explanatory framework of the relationship between mindfulness and learning engagement. Based on this, this study hypothesizes H3: Mindfulness can promote learning engagement by reducing academic burnout.

### 1.4 The serial mediating roles of self-efficacy and academic burnout between mindfulness and learning engagement

While existing research suggests a possible association between mindfulness and academic performance (Verhaeghen, 2023), mechanistic evidence regarding how mindfulness influences learning engagement through specific psychological processes remains relatively limited. Current research has found that self-efficacy and burnout play independent mediating roles between mindfulness and learning engagement (Dai, 2024; Taheri et al., 2019). Additionally, burnout is strongly predicted by self-efficacy, with lower levels of burnout typically being linked to higher levels of self-efficacy (Charkhabi et al., 2013; Cengiz and Peker, 2024). Moreover, meta-analyses have shown that burnout is a significant risk factor affecting students' learning status and academic outcomes, and a significant negative correlation exists between burnout and learning engagement (Madigan and Curran, 2021). These studies suggest that self-efficacy and burnout may constitute a sequential mediating pathway linking mindfulness and learning engagement.

Drawing upon SCT and the SD-R framework, SCT conceptualizes self-efficacy as a belief in one's own capabilities that shapes cognitive appraisals and behavioral persistence (Bandura, 1997), while the SD-R framework views self-efficacy as a personal resource closely linked to how students manage learning demands, academic burnout, and learning engagement (Bakker and Mostert, 2024). The cognitive appraisals and behavioral persistence emphasized by SCT elucidate the specific mechanisms through which self-efficacy functions as a personal resource within the SD-R framework: students with higher self-efficacy are more likely to perceive learning demands as manageable, thereby sustaining their effort and employing adaptive coping strategies; such capability beliefs may mitigate resource depletion, reduce academic burnout, and foster learning engagement. Thus, while SCT delineates the cognitive-motivational functions of self-efficacy, the SD-R framework examines these functions within the context of how students cope with academic demands. Self-efficacy serves as a conceptual bridge between SCT and the SD-R framework, collectively providing an integrative theoretical basis for the serial mediation model proposed in this study. For dance students, mindfulness may enhance self-efficacy by enabling them to address training challenges in a more constructive manner, subsequently alleviating academic burnout and promoting learning engagement. Based on this, this study proposes hypothesis H4: Mindfulness can promote learning engagement by improving self-efficacy and further reducing academic burnout.

**Figure 1:**
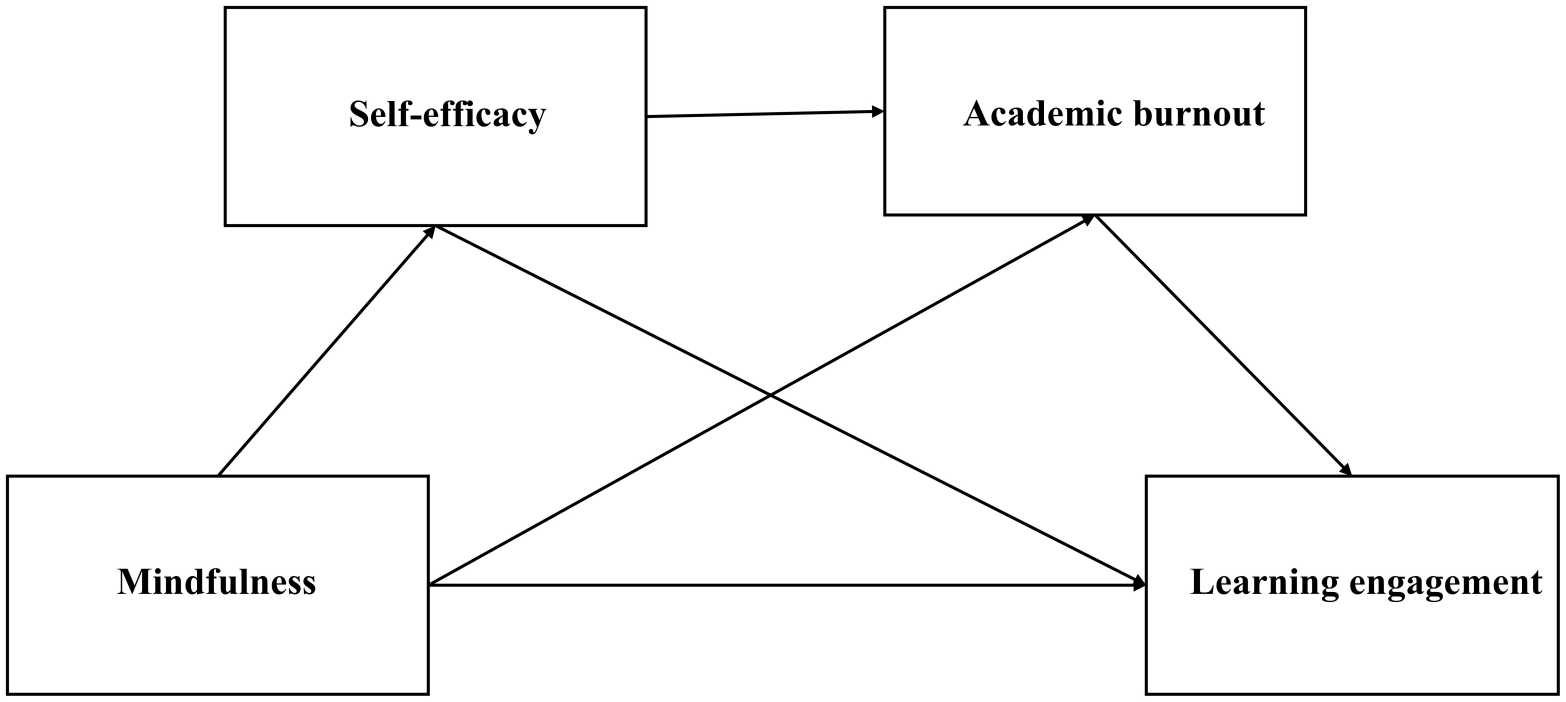
Conceptual model.

This study builds a serial mediation model based on the previously mentioned research (Figure 1).

## 2 Materials and methods

### 2.1 Study design and participants

In compliance with research standards, this study used a stratified convenience sampling method. This study recruited undergraduate dance majors from five universities in five provinces in China (Fujian, Hubei, Jiangxi, Guangxi, and Yunnan). Before participants took part in the survey, they were given a short introduction to its general aim. The study did not disclose the specific research questions. Each participant provided consent prior to completing this questionnaire. Ethical approval was granted by the Ethics Committee of Jimei University (No. 20260312013). A total of 770 surveys were gathered. The requirements for inclusion were: (1) being a current university student, (2) a dance major, and (3) voluntary participation with informed consent. Responses were excluded if they contained obvious missing data, extreme straight-line responses, or other suspicious invalid response patterns. Finally, 711 valid responses were retained (Table 1). They passed data integrity and response quality checks.

**Table 1:** Distribution of demographic variables.

Variable	Category	Frequency	Percentage (%)
Gender	Male	56	8
	Female	655	92
Year	Freshman	108	15
	Sophomore	443	62
	Junior	44	6
	Senior	116	16
Training duration	< 1 h	127	18
	1–3 h	460	65
	3–5 h	87	12
	>5 h	37	5

### 2.2 Measures

#### 2.2.1 Mindfulness scale

Mindfulness was assessed using the Mindful Attention Awareness Scale (MAAS). The MAAS was developed by Brown and Ryan (2003) and revised into Chinese by Chen et al. (2012). The Chinese MAAS contains 15 items, each rated on a 6-point Likert scale ranging from 1 (“almost always”) to 6 (“almost never”). Because the items are negatively worded, they were reverse-scored so that higher mean scores represented greater mindful attention and awareness. Items on the scale include “I rush through activities without being really attentive to them” and “I find it difficult to stay focused on what is happening in the present” (Chen et al., 2012). For the present analysis, the average score across the 15 items was taken as the mindfulness score. Previous studies have confirmed the one-factor structure of the MAAS and supported its reliability and validity in university students from different cultural settings (Brown and Ryan, 2003; Jermann et al., 2009). The scale showed good internal consistency in the current sample (Cronbach's alpha = 0.971).

#### 2.2.2 Self-efficacy scale

In this study, self-efficacy was measured using the General Self-Efficacy Scale (GSES). Schwarzer and Jerusalem (1995) initially created it, and Zhang and Schwarzer (1995) translated it into Chinese. Its Chinese version has also been supported by cross-cultural validation research with first-year university students in Hong Kong. The Chinese GSES contains 10 items and uses a 4-point Likert scale ranging from 1 (“not at all true”) to 4 (“exactly true”). Since all items are positively worded, higher average scores reflect higher levels of self-efficacy. Examples include “If I try hard enough, I can solve difficult problems” and “I can stick to my goals and achieve them.” In the present study, the mean score of the 10 items was calculated as the indicator of self-efficacy. Previous studies have reported that the GSES has good reliability and validity (Zhang and Schwarzer, 1995). The scale showed good internal consistency in the current sample (Cronbach's α = 0.940). Because the GSES assesses a broad, domain-general competence belief, the scores obtained in this study represent students' general confidence in coping with difficult or novel situations rather than self-efficacy specific to academic or dance-learning tasks.

#### 2.2.3 Academic burnout scale

Academic burnout in this study was measured using the Adolescent Student Burnout Inventory (ASBI), developed by Wu et al. (2010). The scale comprises 16 items and assesses three dimensions: physical and mental exhaustion (4 items), academic detachment (5 items), and reduced efficacy (7 items). Each item is rated on a 5-point Likert scale, ranging from 1 (“Strongly Disagree”) to 5 (“Strongly Agree”). Items 1, 4, 7, 11, 14, 15, and 16 are positively worded and are reverse-coded before calculating the total score. After reverse coding, the mean score across all 16 items is computed; a higher score indicates greater overall academic burnout. Sample items include “I can engage in my studies with energy” (reverse-coded) and “I perform poorly in my studies, and I really want to give up.” Although the ASBI was originally developed for elementary and secondary school students, its psychometric properties have since been tested among Chinese college students. In a sample of 660 Chinese college students, confirmatory factor analysis supported its three-factor structure (χ^2^/df = 4.31, RMSEA = 0.07, CFI = 0.91, SRMR = 0.07), with composite reliability coefficients for the three dimensions of 0.73, 0.78, and 0.84, respectively (Luo et al., 2018). These findings support the use of the ASBI among Chinese college students. In this sample, the scale also demonstrated good internal consistency (Cronbach's α = 0.948).

#### 2.2.4 Learning engagement scale

The Utrecht Work Engagement Scale-Student (UWES-S) was used to assess learning engagement. Schaufeli et al. (2002) developed the UWES-S. Fang et al. (2008) translated and revised it into Chinese. Designed to assess Chinese students' positive learning state, the scale comprises 17 items rated on a 7-point Likert scale from 1 (“never”) to 7 (“always/every day”). Higher scores indicate greater learning engagement. Example items include: “When I get up in the morning, I feel like studying,” “I am always enthusiastic about my studies,” and “When I study, I enter a state of deep concentration.” In the Chinese version, both exploratory and confirmatory factor analyses supported a three-factor structure comprising vigor, dedication, and absorption. Internal consistency across dimensions ranged from 0.82 to 0.95. Item loadings ranged from 0.42 to 0.81. Model fit indices were good (Fang et al., 2008). The scale demonstrated good internal consistency in the current sample (Cronbach's α = 0.969).

### 2.3 Data analysis

Tests for common method bias, descriptive statistics, and correlation analysis between variables were conducted in this study using SPSS 26.0. Confirmatory factor analysis was performed using AMOS 24.0. The serial mediation model was tested using the PROCESS macro version 4.1 (Model 6), with 5,000 bootstrap resamples and 95% bias-corrected confidence intervals for indirect effects.

## 3 Results

### 3.1 Common method bias test

Harman's one-factor test was used to assess common method bias. An exploratory factor analysis was conducted in SPSS using all items from the four scales. A total of eight factors with eigenvalues greater than 1 were extracted, and the first factor accounted for 37.2% of the total variance. Since this percentage was below the recommended 40% cutoff, common method bias was not considered a serious issue in the current study.

### 3.2 Confirmatory factor analysis

Confirmatory factor analysis was conducted in AMOS 24.0 to assess the construct validity of the scales. The model fit indices were: χ^2^/df = 2.173, RMSEA = 0.041, CFI = 0.946, TLI = 0.944, SRMR = 0.030. Standardized factor loadings ranged from 0.822 to 0.858 for mindfulness, 0.667 to 0.854 for self-efficacy, 0.699 to 0.750 for academic burnout, and 0.721 to 0.875 for learning engagement. Discriminant validity among the four constructs was also assessed (Table 2), and the results met the criteria, indicating that the four scales have good discriminative power.

**Table 2:** Discriminant validity.

Variable	CR	AVE	1	2	3	4
1. Mindfulness	0.972	0.699	0.836			
2. Self-efficacy	0.940	0.612	0.296^***^	0.782		
3. Learning engagement	0.970	0.652	0.336^***^	0.498^***^	0.808	
4. Academic burnout	0.948	0.532	−0.427^***^	−0.485^***^	−0.569^***^	0.729

The diagonal values represent the square root of AVE; ^***^p < 0.001.

### 3.3 Descriptive statistics and correlation analysis

According to the correlation analysis (Table 3), all variables were well correlated. Mindfulness was positively correlated with self-efficacy (r = 0.29, *p* < 0.01) and learning engagement (r = 0.33, *p* < 0.01), and negatively correlated with academic burnout (r = −0.41, *p* < 0.01). Self-efficacy was positively correlated with learning engagement (r = 0.48, *p* < 0.01) and negatively correlated with academic burnout (r = −0.46, *p* < 0.01). Learning engagement was negatively correlated with academic burnout (r = −0.55, *p* < 0.01). The subsequent mediation study was based on these findings.

**Table 3:** Mean, standard deviation and correlation coefficient of each research variable (*n* = 711).

Variable	M	SD	1	2	3	4
1. Mindfulness	3.84	0.78	1			
2. Self-efficacy	2.51	0.61	0.29^**^	1		
3. Academic burnout	2.78	0.45	−0.41^**^	−0.46^**^	1	
4. Learning engagement	4.49	1.14	0.33^**^	0.48^**^	−0.55^**^	1

^**^P < 0.01.

An independent-samples *t*-test was also used to investigate potential gender differences in mindfulness, learning engagement, self-efficacy, and academic burnout, given the sample's somewhat imbalanced gender distribution. As Table 4 shows, there were no discernible gender differences in mindfulness (t = −0.695, *p* = 0.487), learning engagement (t = 0.502, *p* = 0.616), self-efficacy (t = 1.344, *p* = 0.179), or academic burnout (t = −0.501, *p* = 0.616). This suggests that the unequal gender distribution in the sample had only a limited influence on the main results of this study.

**Table 4:** Independent samples *t*-test for gender differences among variables.

Variable	Male (M ±SD)	Female (M ±SD)	t	*p*
1. Mindfulness	3.77 ± 0.84	3.85 ± 0.77	−0.70	0.49
2. Self-efficacy	2.61 ± 0.62	2.50 ± 0.61	1.34	0.18
3. Academic burnout	2.75 ± 0.46	2.78 ± 0.45	−0.50	0.62
4. Learning engagement	4.56 ± 1.14	4.48 ± 1.14	0.50	0.62

### 3.4 Regression analysis

Based on the regression results from PROCESS Model 6 (Table 5), mindfulness significantly positively predicted self-efficacy (β = 0.29, t = 8.07, *p* < 0.001) and significantly negatively predicted academic burnout (β = −0.30, t = −9.16, *p* < 0.001). Self-efficacy also significantly negatively predicted academic burnout (β = −0.37, t = −11.30, *p* < 0.001). Mindfulness significantly positively predicted learning engagement (β = 0.09, t = 2.79, *p* = 0.005), and self-efficacy also had a significant positive effect on learning engagement (β = 0.28, t = 8.19, *p* < 0.001). By contrast, academic burnout significantly negatively predicted learning engagement (β = −0.39, t = −10.87, *p* < 0.001), showing the strongest predictive effect among the three predictors.

**Table 5:** Regression modeling.

Item	Self-efficacy		Academic burnout		Learning engagement	
	β	t	β	t	β	t
Mindfulness	0.29	8.07^***^	−0.30	−9.16^***^	0.09	2.79^**^
Self-efficacy			−0.37	−11.30^***^	0.28	8.19^***^
Academic burnout					−0.39	−10.87^***^
R	0.29		0.54		0.61	
R^2^	0.08		0.30		0.38	
F	65.17		148.26		141.26	

^**^p < 0.01; ^***^p < 0.001.

**Figure 2:**
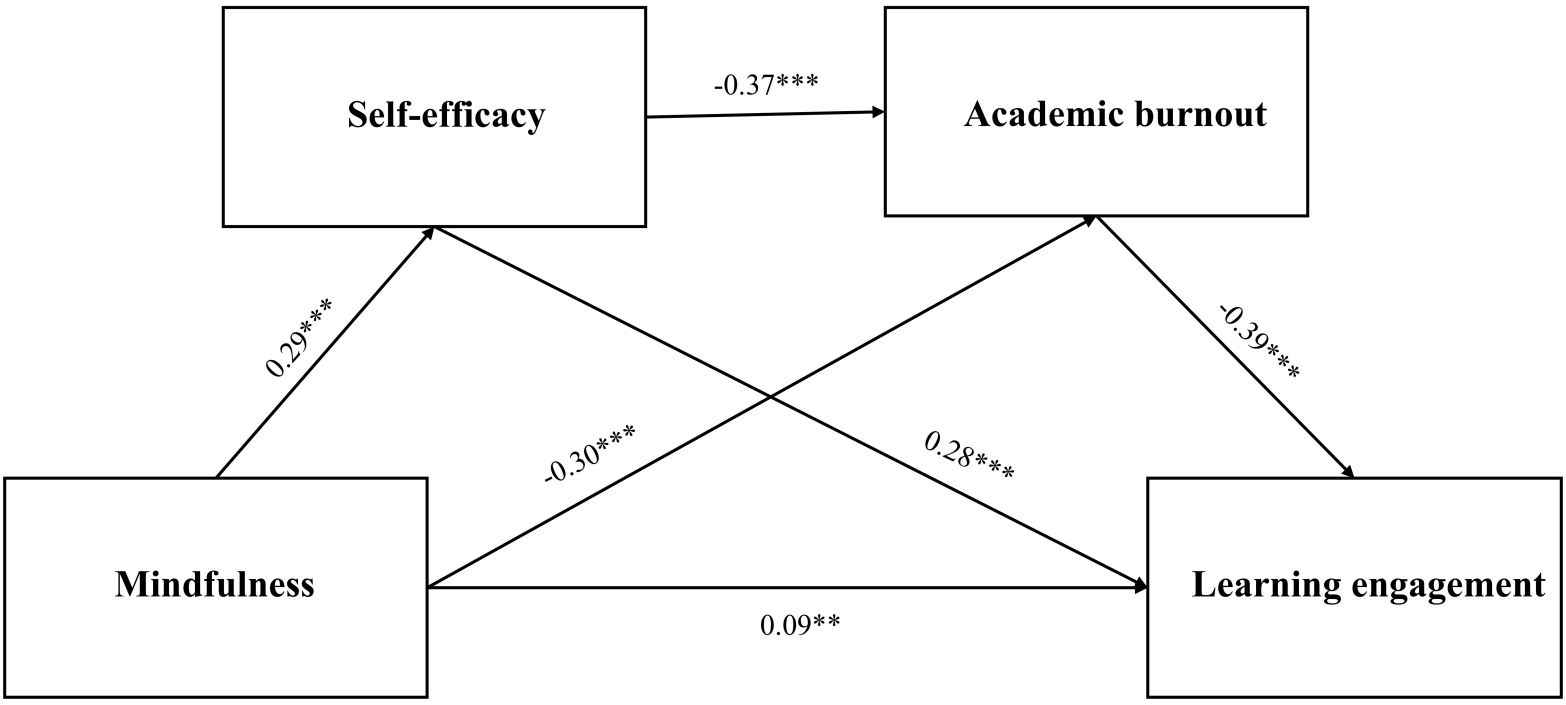
The serial mediation model. **p < 0.01; ***p < 0.001.

Overall, the serial mediation model demonstrated satisfactory explanatory power. The R^2^ values for predicting self-efficacy, academic burnout, and learning engagement were 0.08, 0.30, and 0.38, respectively. The corresponding F values were 65.17, 148.26, and 141.26. All F-tests were statistically significant (*p* < 0.001). Figure 2 presents the standardized path coefficients among mindfulness, self-efficacy, academic burnout, and learning engagement.

### 3.5 Serial mediation analysis

Using a serial mediation model, the mediating roles of self-efficacy and academic burnout in the relationship between mindfulness and learning engagement were investigated. Confidence intervals were estimated using bootstrap techniques to test the significance of the overall effect, the direct effect, and each indirect effect. This analysis bootstrapped 5,000 resamples and calculated a 95% confidence interval (Alfons et al., 2022). When an effect's 95% bootstrap confidence interval excluded zero, it was deemed significant.

According to Table 6, the overall impact of mindfulness on learning engagement was 0.48, with a 95% confidence interval [0.38, 0.58]. Because the interval excluded zero, this indicates a significant total effect. After including the mediators, the direct influence of mindfulness on learning engagement was 0.13. The bootstrap 95% confidence interval was 0.04 to 0.23. This interval also excluded zero, indicating that the direct influence was significant. These results imply that mindfulness may have an indirect impact through a variety of mediating pathways, in addition to being a direct predictor of learning engagement.

**Table 6:** Mediation variable test.

Impact pathways	Effect	se	t	*p*	LLCI	ULCI	Relative effect value (%)
Total effect	0.48	0.05	9.31	0.000	0.38	0.58	100.00
Direct effect	0.13	0.05	2.79	0.005	0.04	0.23	27.80
Ind1	0.12	0.02			0.07	0.17	24.27
Ind2	0.17	0.03			0.12	0.22	35.27
Ind3	0.06	0.01			0.04	0.09	12.66

The bootstrap confidence intervals for all three indirect paths did not include zero, indicating that all indirect effects were significant. The first route involved self-efficacy as a single mediator (mindfulness → self-efficacy → learning engagement). The indirect effect was 0.12, with a 95% bootstrap interval of 0.07 to 0.17. This indirect path accounted for 24.27% of the total effect. These results indicate that self-efficacy significantly mediates the relationship between mindfulness and learning engagement, thereby supporting Hypothesis 2.

The second pathway was a single mediation through academic burnout (mindfulness → academic burnout → learning engagement). The indirect effect was 0.17, with a 95% bootstrap interval of 0.12 to 0.22. This indirect path accounted for 35.27% of the total effect. These findings confirm Hypothesis 3, showing that academic burnout significantly mediates the relationship between mindfulness and learning engagement.

The third pathway was serial mediation (mindfulness → self-efficacy → academic burnout → learning engagement). The indirect effect was 0.06, with a 95% confidence interval of [0.04, 0.09]. This pathway accounted for 12.66% of the total effect. These results suggest that mindfulness may first increase self-efficacy, then reduce academic burnout, and finally promote learning engagement. These results support Hypothesis 4.

In summary, the present findings supported all four hypotheses.

## 4 Discussion

From an integrated perspective of SD-R theory and SCT, this study confirmed the role of mindfulness in learning engagement and the associated pathways in a high-standard dance learning context. The results show that mindfulness can directly improve learning engagement and also operates through a serial mediation pathway in which mindfulness increases self-efficacy, which then reduces academic burnout and ultimately promotes learning engagement. These findings offer empirical support for understanding how dance majors maintain a positive learning state in ongoing training contexts, and they also offer useful implications for psychological support and intervention in dance education.

### 4.1 Mindfulness and learning engagement

The results of this study indicate that mindfulness significantly predicts learning engagement among dance students, largely consistent with existing research findings. Egbaria (2024) found a significant positive correlation between mindfulness and learning engagement in a sample of college students, with positive relationships observed across dimensions including observation, description, non-judgement, interaction with internal experience, and conscious action. Zhao et al. (2024) found that “acting with awareness” was the strongest predictor of learning engagement. Furthermore, Hammill et al. (2023)'s intervention study confirmed that mindfulness helps improve university students' self-regulation abilities, thereby promoting learning engagement. All this evidence demonstrates a stable positive link between mindfulness and learning engagement.

However, the direct effect of mindfulness on learning engagement is not consistently strong across studies. Aldbyani et al. (2025) found a positive bivariate correlation between learning engagement and mindfulness, but after adding “the existence of a sense of professional mission” and “the exploration of a sense of professional mission” to the mediation model, the direct effect of mindfulness on learning engagement became non-significant. This suggests that the effect of mindfulness on learning engagement may rely more on mediating mechanisms in certain contexts than on a simple direct effect. Therefore, this study found that mindfulness can still directly predict learning engagement among dance students. This may indicate that in training environments characterized by high intensity, high repetition, high-precision error correction, and high exposure to evaluation, the supportive effects of mindfulness on attentional stability, emotion regulation, and training persistence are more directly reflected in learning engagement. This is also supported by empirical studies on Australian dance students and Indian classical dancers, where mindfulness training helps enhance the psychological resources of dance learners, buffer stress in training situations, and provide psychological support for sustained engagement (Blevins et al., 2022; Pandya, 2025).

From the perspective of attention regulation, mindfulness may help individuals reduce automatic, stimulus-driven responses and sustain awareness of the present-moment experience (Brewer et al., 2013; Hölzel et al., 2011). This may help students focus more steadily on movement execution and bodily feedback during dance training, thereby reducing the disruptive effects of distraction and rumination on the training process. Mindfulness can promote positive reappraisal in stressful situations, enabling individuals to understand negative events in a more open, less defensive manner and to adjust their cognitive and behavioral responses accordingly (Garland et al., 2009). Therefore, students may be less likely to view fatigue, mistakes, and repeated corrections as evidence of incompetence, and more likely to see them as modifiable information in the training process, thus maintaining their willingness to participate in training.

Therefore, this study not only supports the positive relationship between mindfulness and learning engagement, but also suggests that, in the high-demand training context of dance, the promoting effect of mindfulness on learning engagement may have greater explanatory power than in general learning situations.

### 4.2 The mediating effect of self-efficacy

The results indicate that self-efficacy plays a significant mediating role between mindfulness and learning engagement, supporting Hypothesis 2. This suggests that the positive effect of mindfulness on learning engagement is not only direct but also operates through enhanced students' self-assessment of their abilities. This result is largely in line with earlier studies (Dai, 2024), and Abdel Hadi and Al-Quraan (2025) also found a strong positive correlation between self-efficacy and mindfulness. Furthermore, Wang and Zhang (2024) and Woreta et al. (2025) demonstrate that self-efficacy is an important psychological variable predicting learning engagement. Together, these studies corroborate the research's findings, showing that mindfulness affects learning engagement not only by enhancing emotional states but, more significantly, by boosting students' self-confidence and sense of control over tasks, which can further encourage sustained student engagement in learning activities.

In the learning process of dance majors, students often need to invest sustained time, energy, and physical effort before seeing significant progress. Therefore, maintaining learning engagement depends not only on the student's willingness to invest but also on their continued belief in their ability to succeed. Only when students maintain basic confidence in their abilities are the attentional stability, emotional regulation, and cognitive openness brought about by mindfulness more likely to translate into sustained learning engagement. This finding is broadly consistent with the core proposition of SCT that individuals with higher self-efficacy are more likely to take on challenging tasks and persist in the face of setbacks.

### 4.3 The mediating effect of academic burnout

The findings support Hypothesis 3 by showing that burnout significantly mediates the relationship between mindfulness and learning engagement, even on its own. This finding is consistent with existing research (Mostafavi et al., 2024). Notably, among the multiple mediation pathways examined in this study, the indirect effect via burnout accounted for the highest proportion (35.27%). However, it remains unclear whether this effect is consistently higher than those of other pathways. However, in the demanding and highly engaging training context of dance, burnout may be the most critical energy-expenditure variable in the process by which mindfulness influences the learning engagement of dance students, and this mechanism may have greater explanatory significance in the context of dance learning.

This finding may be linked to the high-intensity context of dance learning. For dance students, the decline in learning engagement does not necessarily stem primarily from a lack of motivation. However, it is more likely due to the physical and mental exhaustion resulting from continuous training. When students are chronically fatigued, emotionally exhausted, and alienated from learning, classroom participation, sustained practice, and focused engagement are likely to decline.

Existing research also supports this explanation. A meta-analysis found a significant negative correlation between mindfulness and academic burnout and emotional exhaustion (Cai et al., 2025b), indicating that higher levels of mindfulness reduce the likelihood of students experiencing fatigue and exhaustion during the learning process. Further research revealed that academic burnout is not simply a negative outcome; it often further erodes students' energy, focus, and willingness to continue participating in learning (Sun et al., 2025). This suggests that mindfulness, academic burnout, and learning engagement collectively constitute an intrinsically linked pathway. Therefore, this study's contribution is to elevate academic burnout from a negative outcome variable to a key explanatory variable for understanding the formation mechanism of learning engagement among dance students.

This outcome closely aligns with the SD-R theory's energy-depletion pathway. The theory posits that sustained learning demands induce fatigue, alienation, and inefficiency through resource exhaustion, while personal resources can buffer this depletion (Salmela-Aro and Upadyaya, 2014). Dance learning is characterized by high physical load and continuous practice, and sustained engagement primarily depends on relatively stable physical and mental energy and emotional regulation. Once students experience persistent fatigue, emotional exhaustion, or psychological withdrawal, they tend to quickly reduce training engagement, decrease classroom participation, and exhibit avoidance of practice and inattentiveness, directly weakening their learning engagement. Conversely, when mindfulness helps students reduce negative, repetitive thinking and emotional involvement, and understand the tension, fatigue, and frustration of training in a more constructive way, their sense of exhaustion and burnout often improves first. Therefore, in the context of dance learning, reducing academic burnout may be a more direct and significant pathway through which mindfulness promotes learning engagement.

In conclusion, given the contextual characteristics of dance training, it is reasonable that the indirect effects of academic burnout account for the largest proportion. Therefore, given the heavy workload of dance learning, a mindfulness intervention programme with the core objectives of reducing burnout and preserving psychological energy may be more suitable for the actual needs of dance students.

### 4.4 The serial mediating effect of self-efficacy and academic burnout

The findings support a sequential mechanism of “mindfulness → self-efficacy → academic burnout → learning engagement,” showing that self-efficacy and academic burnout have a significant serial mediation effect in the link between mindfulness and learning engagement. In general, this result is in line with earlier studies on the correlations between these variables. This finding suggests that mindfulness's impact on learning engagement is not only direct but may also occur through a sequential process that first enhances positive competence beliefs such as self-efficacy and then buffers the exhaustion response associated with academic burnout.

Prior studies have demonstrated a strong negative correlation between academic burnout and its components, such as diminished efficacy and emotional weariness, and self-efficacy (Charkhabi et al., 2013). This implies that fatigue and disengagement during learning are less common among students with higher self-efficacy. These results provide further evidence for the role of self-efficacy in lowering academic burnout, suggesting that self-efficacy may both prevent negative burnout reactions and encourage positive learning states.

By integrating SD-R and SCT, mindfulness, as a positive personal resource, helps dance students stabilize attention, reduce rumination and negative self-evaluation, and become less prone to self-doubt when facing tension, fatigue, and setbacks in training. Instead, they are more likely to refocus on manageable movement points and process goals, thereby enhancing their self-efficacy (González-Martín et al., 2023; Schunk and DiBenedetto, 2021). Higher self-efficacy can enhance students' sense of control and confidence in coping with tasks, helping to reduce perceived threat and emotional exhaustion under continuous learning demands, thereby lowering levels of academic burnout (Charkhabi et al., 2013; Cengiz and Peker, 2024). As academic burnout decreases, students retain more psychological energy for classroom participation, sustained practice, and task focus, ultimately exhibiting higher levels of learning engagement. In other words, the serial pathway revealed in this study essentially reflects a continuous psychological process from personal resource building to exhaustion buffering and then to increased engagement.

The significance of this study lies not only in reaffirming the pairwise correlations between these variables, but also in revealing that, in the demanding training context of dance students, self-efficacy and burnout do not operate in parallel on learning engagement, but rather likely work together in a sequential manner. The positive effects of mindfulness do not merely stem from feeling more stable or confident, but rather from first enhancing students' judgement of their own abilities and the controllability of their training. This belief in competence then weakens fatigue and alienation, ultimately translating into a higher level of learning engagement. Compared with general learning situations, this serial pathway is more explanatory for dance students, because dance learning not only emphasizes skill mastery but also relies heavily on students maintaining mental and physical energy and training perseverance through repeated practice, continuous error correction, and exposure to evaluation.

This finding not only expands the explanation of the mechanism by which mindfulness influences learning engagement but also suggests that in dance education practice, simply emphasizing the enhancement of motivation may not be sufficient. It is more important to focus simultaneously on establishing students' belief in competence and on preventing the risk of burnout, thereby more effectively supporting their sustained learning engagement.

## 5 Practical and theoretical implications

This study integrates SD-R and SCT, using the high-standard learning environment of undergraduate dance students as a backdrop, to explain how students sustain their learning engagement under long-term pressure and high demands. It further explores the internal mechanism through which mindfulness operates in this process, mediated by self-efficacy and academic burnout, thereby constructing and verifying the sequential mechanism of “mindfulness → self-efficacy → academic burnout → learning engagement.” The findings deepen current understanding of the relationship between mindfulness and learning engagement, provide a clearer path for explaining how psychological resources are transformed into sustained engagement in a professional training environment, and fill a gap in relevant evidence in the field of dance education. In addition, the study offers useful theoretical support for subsequent educational assistance and intervention design. To strengthen dance students' sustained engagement in class and rehearsal, support strategies should not stop at emotional comfort or simple encouragement of willpower. Instead, mindfulness training, the systematic development of self-efficacy, and the management of academic burnout should be combined into an integrated approach. In this way, students' motivational resources can be strengthened while unnecessary energy loss is reduced, which may further support more stable and lasting learning development.

## 6 Limitations

There are still certain restrictions on this study. First, the results show more theoretical association routes between variables than rigorously established causal linkages, due to the online cross-sectional questionnaire design and the mediation effect test based on the theoretical concept of serial mediation. Future studies could examine temporal order and causal links more clearly by using longitudinal or experimental designs. Secondly, despite sufficient statistical power, there may be selection bias and insufficient regional representation—especially considering the regional development differences in China and the diversity of university types. The universality of the research results across a wider range of regions and institutional types needs to be further verified. The generalizability of these findings may be strengthened by including comparative samples from other provinces and universities. Third, there is a significant disparity in the gender composition of the sample, with females accounting for 92% and males for only 8%. This ratio reflects the reality that dance majors are predominantly female. The limited number of male participants may affect the applicability of the findings to male dance students. The results likely reflect the psychological characteristics and academic performance of female students, and it remains unclear whether the observed serial mediation effect applies equally to male students. Although independent samples *t*-tests revealed no significant differences between male and female students regarding the four study variables, this indicates only that there were no significant differences in mean levels; it does not imply that the direct and indirect effects within the model are identical across male and female student groups. Therefore, caution should be exercised when generalizing these findings to male dance majors. Future research could further expand the sample size of male students to achieve a more balanced gender ratio and employ moderated mediation analysis to examine further whether gender differences exist in this serial mediation mechanism.

## 7 Conclusions

This study examined the link between mindfulness and learning engagement, along with its related pathways, among undergraduate dance majors. The results showed a significant serial mediation effect through self-efficacy and academic burnout between mindfulness and learning engagement. Mindfulness not only directly and positively predicted learning engagement, but also indirectly promoted it by increasing self-efficacy and then reducing academic burnout. The results also supported the independent mediating effects of self-efficacy and academic burnout, as well as their sequential mediation effect. Overall, this study took an integrated view of the motivational process and the energy-depleting process of personal resources. This study identified key psychological mechanisms that influence learning engagement in a high-standard dance training context. These findings provide theoretical support and practical implications for improving dance majors' learning engagement through mindfulness training and resource-strengthening strategies.

## Data Availability

The raw data supporting the conclusions of this article will be made available by the authors, without undue reservation.
